# SG-APSIC1065: The effectiveness of an ultraviolet-C device for terminal room disinfection in an intensive care unit

**DOI:** 10.1017/ash.2023.35

**Published:** 2023-03-16

**Authors:** Zhi-Yuan Shi, Pei-Hsuan Huang, Chun-Hsi Tai, Hsin-Yi Hung, Yuh-Feng Chen, Ying-chun Chen, Hui-Mei Huang

**Affiliations:** Taichung Veterans’ General Hospital, Taichung, Taiwan; Taichung Veterans’ General Hospital, Taichung, Taiwan; Taichung Veterans’ General Hospital, Taichung, Taiwan; Taichung Veterans’ General Hospital, Taichung, Taiwan; Taichung Veterans’ General Hospital, Taichung, Taiwan; Taichung Veterans’ General Hospital, Taichung, Taiwan; Taichung Veterans’ General Hospital, Taichung, Taiwan

## Abstract

**Objectives:** Medical devices and the hospital environment can be contaminated easily by multidrug-resistant bacteria. The effectiveness of cleaning practices is often suboptimal because environmental cleaning in hospitals is complex and depends on human factors, the physical and chemical characteristics of environment, and the viability of the microorganisms. Ultraviolet-C (UV-C) lamps can be used to reduce the spread of microorganisms. We evaluated the effectiveness of an ultraviolet-C (UV-C) device on terminal room cleaning and disinfection. **Methods:** The study was conducted at an ICU of a medical center in Taiwan. We performed a 3-stage evaluation for the effectiveness of UV-C radiation, including pre–UV-C radiation, UV-C radiation, and a bleaching procedure. The 3 stages of evaluation were implemented in the ICU rooms from which a patient had been discharged or transferred. We collected the data from adenosine triphosphate (ATP) bioluminescence testing, colonized strains, and their corresponding colony counts by sampling from the environmental surfaces and air. We tested 8 high-touch surfaces, including 2 sides of bed rails, headboards, footboards, bedside tables, monitors, pumping devices, IV stands, and oxygen flow meters. **Results:** In total, 1,696 environmental surfaces and 72 air samples were analyzed. The levels of ATP bioluminescence and colony counts of isolated bacteria decreased significantly after UV-C radiation and bleaching disinfection for both the environmental and air samples (*P* < .001). Resistant bacteria (vancomycin-resistant *Enterococcus*, VRE) were commonly isolated on the hard-to-clean surfaces of monitors, oxygen flow meters, and IV pumps. However, they were also eradicated (*P* < .001). **Conclusions:** UV-C can significantly reduce environmental contamination by multidrug-resistant microorganisms. UV-C is an effective device to assist staff in cleaning the hospital environment.

